# Chromosome-Level Assemblies of the Allohexaploid Genomes of *Conyza sumatrensis* and *Conyza bonariensis*

**DOI:** 10.1093/gbe/evaf065

**Published:** 2025-04-04

**Authors:** Anthony Côrtes Gomes, Jacob Montgomery, André Lucas Simões Araujo, Sarah Morran, Luan Cutti, Eric Patterson, Sofia Marques Hill, Maor Matzrafi, Anil Shrestha, Aldo Merotto, Fatemeh Abdollahi, David R Nelson, Victor Llaca, Kevin Fengler, Camila Ferreira de Pinho, Todd A Gaines

**Affiliations:** Crop Science Department, Federal Rural University of Rio de Janeiro, Rio de Janeiro, Brazil; Department of Agricultural Biology, Colorado State University, Fort Collins, CO, USA; Department of Agricultural Biology, Colorado State University, Fort Collins, CO, USA; Department of Agricultural Biology, Colorado State University, Fort Collins, CO, USA; Department of Plant, Soil and Microbial Sciences, Michigan State University, East Lansing, MI, USA; Department of Plant, Soil and Microbial Sciences, Michigan State University, East Lansing, MI, USA; Department of Agricultural Biology, Colorado State University, Fort Collins, CO, USA; Department of Plant Pathology and Weed Research, Newe Ya’ar Research Center, Agricultural Research Organization, Volcani Institute, Yishay, Israel; Department of Plant Science, California State University, Fresno, CA, USA; Department of Crop Sciences, Federal University of Rio Grande do Sul, Porto Alegre, Brazil; Department of Agricultural Biology, Colorado State University, Fort Collins, CO, USA; Department of Microbiology, Immunology and Biochemistry, University of Tennessee Health Science Center, Memphis, TN, USA; Genome Center of Excellence, Corteva Agriscience, Johnston, IA, USA; Genome Center of Excellence, Corteva Agriscience, Johnston, IA, USA; Crop Science Department, Federal Rural University of Rio de Janeiro, Rio de Janeiro, Brazil; Department of Agricultural Biology, Colorado State University, Fort Collins, CO, USA

**Keywords:** *Conyza*, *Erigeron*, weed genomics, genome assemblies, synteny, phylogenetic

## Abstract

*Conyza sumatrensis* and *Conyza bonariensis* are two important hexaploid weed species that impact crop production systems across the globe. Here, we report reference genome assemblies for both species. The sequenced accession of *C. sumatrensis* is resistant to multiple herbicides and was collected from the state of Paraná in Brazil, while the accession of *C. bonariensis* was collected from California, USA. Genomic long read data was used along with optical mapping data to assemble the *C. sumatrensis* genome into a single haplome at chromosome-level contiguity. The same approach was used along with chromatin contact mapping data to phase the haplotypes of *C. bonariensis* and generate two chromosome-level haplome assemblies. Subgenome-specific sequences were identified and used to classify the three subgenomes within each assembly. The assemblies are highly complete based on the presence of conserved single-copy orthologs and telomeres, and the size of these assemblies agrees with previous flow cytometry estimates. Full-length transcript sequencing along with gene models from other Asteraceae members was used to predict gene models within each assembly. The genomic resources reported here will be useful for investigations into evolutionary and ecological questions around weed invasion and management for these two species.

**Significance**

This study reports the first reference genome assemblies of two weed species that are important to agriculture due to their impact on crop production systems. The genomes reported in this study will be utilized to investigate the biology and evolution of *Conyza sumatrensis* and *Conyza bonariensis*.

## Introduction

Within Asteraceae, the *Conyza* (syn. *Erigeron*) genus contains several species including *Conyza bonariensis* (hairy fleabane) and *Conyza sumatrensis* (Sumatran fleabane), that are important pests of agricultural production systems (Al-Qthanin et al. 2024; Kalsing et al. 2024). Within the *Conyza* genus, *Conyza canadensis* (Canada fleabane) is the most prominent globally, and is a common pest of diverse agronomic settings (Weaver 2001). *Conyza bonariensis* and *C. sumatrensis* are morphologically similar and are less prevalent in North America than *C. canadensis* (Florentine et al. 2021). Both species are annual or short-lived perennial and invasive to agricultural and nonagricultural habitats worldwide (Prieur-Richard et al. 2000; Okumu et al. 2022). These species form a basal rosette over winter or spring and produce seeds in the following season that are dispersed widely by wind. *Conyza* species are prolific seed producers, with *C. bonariensis* plants producing up to 120,000 seeds per plant (Wang et al. 2018). The seeds do not have a long dormancy requirement. Under adequate moisture, 50% of *C. bonariensis* seeds harvested from a mature plant germinated the same day (Shrestha et al. 2022).

Different populations of both *C. bonariensis* and *C. sumatrensis* species have evolved simple, cross, or multiple resistance to many herbicides used to control them (Heap 2024). To date, cases of resistance have been reported for glyphosate, 2,4-D, paraquat, diuron, saflufenacil, and penoxsulam, among other herbicides with different modes of action (Leal et al. 2022; Heap 2024). In 2017, in the state of Paraná, Brazil, one of the most complex cases of resistance was observed for a *C. sumatrensis* resistant population, with resistance to herbicides with five different sites of action: EPSPS inhibitors, photosystem II inhibitors, photosystem I inhibitors, protoporphyrinogen oxidase inhibitors, and auxin mimics (Pinho et al. 2019). Some Brazilian populations of *C. sumatrensis* have been shown to have a nontarget site resistance mechanism for the auxin mimic 2,4-D in which a rapid cell death response is induced following treatment that limits herbicide mobility in the plant (Queiroz et al. 2020; Souza et al. 2023).

Ploidy level varies across the *Conyza* genus (Garcia et al. 2013). *Conyza canadensis* has a relatively small, diploid genome that was among the first genomes of weedy species to be assembled to chromosome-level (Laforest et al. 2020). While ploidy may vary for *C. bonariensis*, it and *C. sumatrensis* are generally considered hexaploid (Garcia et al. 2013; Soares et al. 2015). Despite differences in ploidy levels that could preclude gene flow, evidence suggests that alleles have been exchanged at low frequency between diploid *C. canadensis* and hexaploid *C. bonariensis* (Okada et al. 2015; Soares et al. 2015). The hexaploid species *C. bonariensis* and *C. sumatrensis* are inter-fertile and capable of producing hybrids resulting in gene flow of herbicide-resistant alleles (Kalsing et al. 2024). Genetic studies have revealed substantial within- and between-population diversity for these species, driven in part by self-compatible reproductive systems and wind-mediated seed dispersal (Okada et al. 2015; Qasem et al. 2023; Kalsing et al. 2024).

A recent collaborative effort has led to the production of reference genome assemblies for many nonmodel weed species from around the world (Montgomery et al. 2024). The availability of these resources for other weed species has enabled elucidation of the genetic basis for several traits, including sex, herbicide resistance, and agricultural management, more broadly (Montgomery et al. 2021; Kreiner et al. 2022; Johnson et al. 2025). Here, we report reference genome assemblies for *C. bonariensis* and *C. sumatrensis*. These chromosome-level assemblies are highly complete with gene model annotations. These tools will be foundational to genomic investigations into these species to develop improved management practices. Additionally, genomic approaches may be used to identify synthesis pathways of medicinally valuable metabolites produced by these species (Mahanur et al. 2023).

## Results and Discussion

### Genome Assembly

We generated 67.8 Gbp (estimated 1C coverage = 31.5X) and 92.7 Gbp (estimated 1C coverage = 45.5X) of genomic PacBio HiFi data for *C. sumatrensis* and *C. bonariensis*, respectively. The 1C genome size estimations for *C. bonariensis* (2.039 Gbp) and *C. sumatrensis* (2.152 Gbp) were derived from flow cytometry reported by Garcia et al. (2013). For *C. bonariensis*, we also generated 81 Gbp of Hi-C data. For *C. sumatrensis*, assembly of HiFi data alone resulted in good contiguity in our primary assembly, and the resulting contigs were scaffolded using Bionano Optical maps to form a single haplotype-collapsed chromosome-level assembly (Table 1). The *C. sumatrensis* individual used for the assembly was from an inbred line, enabling the assembly of a single haplotype. In the case of *C. bonariensis*, Hi-C data was integrated along with HiFi data to generate the primary assembly. The inclusion of Hi-C data allowed us to phase the contigs into two haplomes. These phased contigs were scaffolded using Bionano maps, and the resulting hybrid scaffolds were assembled into chromosomes using Hi-C contact information. This process resulted in two haplome assemblies for *C. bonariensis*. All assemblies are near the expected genome size, chromosome-level, and highly complete based on the presence of Benchmarking Universal Single-Copy Orthologs (BUSCOs) and telomeres (Table 1; supplementary tables S1 and S2, Supplementary Material online).

**Table 1:** Summary statistics for the genome assemblies of *C. sumatrensis* and each haplotype of *C. bonariensis*

	*C. sumatrensis*	*C. bonariensis* Hap1	*C. bonariensis* Hap2
Assembly length (Mbp)	2,053	1,925	1,894
Estimated genome size (Mbp)^a^	2,152	2,039	2,039
N's per 100 kbp	26.1	27.1	3.21
Number of chromosomes	27	27	27
Number of contigs	55	115	112
Chromosome N50 (Mbp)	75.3	72.8	72.1
Contig N50 (Mbp)	70.3	55.7	58.1
Number of gene models	90,322	97,866	96,108
Percent complete genome BUSCO	99.2	99.1	99.1
Percent complete transcript BUSCO	96.6	96.9	96.5

^a^Estimated by Garcia et al. (2013).

### Subgenome Assignment, Genome Annotation, and Synteny Analysis

Ploidy level across members of *Conyza* is variable (Garcia et al. 2013); however, the genomes of both species used in this study assembled into 27 haploid chromosomes, indicating each plant was hexaploid (1*x* = 9, *n* = 27). Subgenome-specific kmers were identified within each species and used to assign each chromosome to one of three subgenomes within each assembly.

The genomes of *C. sumatrensis* and *C. bonariensis* are rich with repetitive elements, with 66% to 70% of each genome being annotated as repetitive content including 38% to 40% retroelements with LTR elements forming the largest retroelement group (supplementary table S3, Supplementary Material online). To annotate gene models within each genome assembly, we generated 4.6 and 13.3 Gbp of full transcript PacBio isoseq data for *C. sumatrensis* and *C. bonariensis*, respectively. This data was used along with protein sets from other Asteraceae members to generate gene sets for each genome containing 90,322 and 97,866 total genes for *C. sumatrensis* and *C. bonariensis*, respectively. At the whole genome level, duplicated BUSCOs were ≥96% (supplementary table S2, Supplementary Material online), which was expected given these species are both hexaploid. BUSCO duplication within a single subgenome is ≤3.3% for all assemblies, while single-copy BUSCO scores range from 85.3% to 94.6% within subgenome (supplementary table S2, Supplementary Material online). Interestingly, the A subgenome has a higher complete BUSCO score (97.7% for *C. sumatrensis* and 97.9% for *C. bonariensis* haplotype 1) than either B (89.3% and 90.6%) or C (89.6% and 90%) for each assembly. While we cannot be certain that some genes were simply not assembled and/or annotated in subgenomes B and C of both species, we propose the hypothesis that the apparent loss of genes within subgenomes B and C is due to deletions of genomic sections, a process that accounts for gene loss following polyploidization events in the evolution of other polyploid plant lineages (Cheng et al. 2018; Li et al. 2024).

**Fig. 1.**
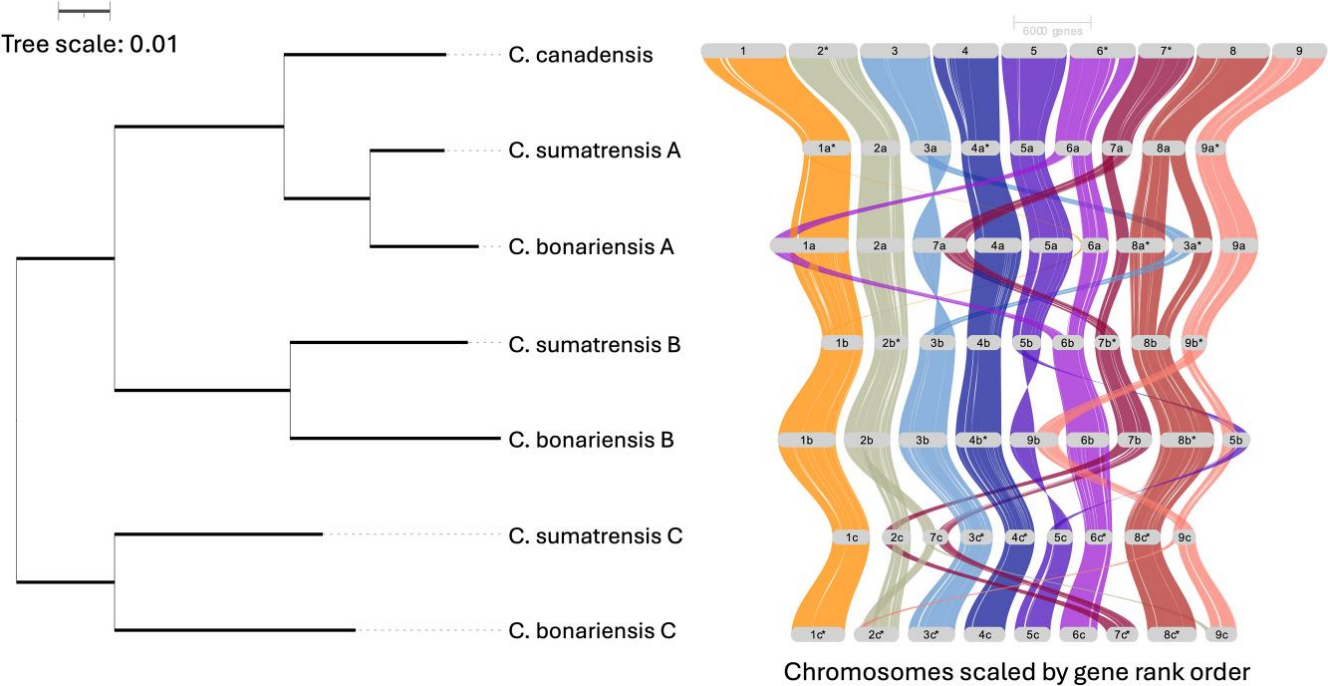
Relatedness and comparison of genome structure between *C. canadensis* and the subgenomes of *C. sumatrensis* and the haplotype 1 assembly of *C. bonariensis*. Phylogenetic tree on the left generated using orthofinder based on all orthologous protein sequences across each genome/subgenome with branch length representing substitutions per site. Ribbon plot on the right represents synteny of coding regions generated by GENESPACE. An asterisk following a chromosome name indicates that chromosome was inverted in the plot for clarity. Ribbons are colored based on where they originate in the *C. canadensis* assembly.

Phylogenetic clustering of subgenomes supports the subgenome assignments, and comparison of synteny across each subgenome shows several large-scale genomic rearrangements in each species (Fig. 1). Phylogenetic analysis suggests that the A subgenome from each assembly is most related to *C. canadensis*, another agronomically important pest in agricultural settings (Laforest et al. 2020). Thus, *C. canadensis* or some close relative may be the A subgenome progenitor for this species complex.

### Identification, Nomenclature, and Classification of Cytochrome P450 Monooxygenase Genes

*Cytochrome P450 monooxygenase* (*CYP*) genes participate in secondary metabolism and can be involved in pesticide resistance mechanisms and other important traits (Gaines et al. 2020). Thus, this diverse gene family is useful to assess the completeness of the genome resources and of particular interest to molecular studies into herbicide resistance and secondary metabolite production. A total of 810 and 865 *CYP* candidate genes were identified from *C. bonariensis* and *C. sumatrensis*, respectively, which were named by the *CYP* International Nomenclature Commission. These candidates were categorized into 47 and 48 *CYP* families and various subfamilies, respectively. Detailed information on the families, subfamilies, and gene IDs of all identified *CYP* genes is provided in supplementary tables S4 to S6, Supplementary Material online. Among the candidates, 518 and 585 full-length *CYP* genes were identified in *C. bonariensis* and *C. sumatrensis*, respectively. These genes, ranging from 350 to 600 amino acids in length, were used for clan classification analysis. The full-length *CYP* genes were grouped into two major clades: the A-type clade, which includes clan 71, and the non-A-type clade, comprising eight clans (clans 51, 74, 710, 85, 72, 711, 97, and 86). Some clans, such as clans 51, 74, 97, 710, and 711, consisted of only one gene family each, while others (clans 71, 72, 85, and 86) encompassed multiple gene families (supplementary fig. S1a and b, Supplementary Material online).

## Materials and Methods

### Plant Material

In 2017, seed of *C. sumatrensis* was collected from a crop production area near Assis Chateaubriand, Paraná, Brazil. This area had been managed with herbicides, and the population is resistant to several herbicides (Pinho et al. 2019). This seed was imported to Colorado, USA, and to comply with the Convention on Biological Diversity, the collection was registered with Brazil SISGEN under number A05A40D. An ABS Annex is on file and available upon request to the authors. Individuals from the *C. sumatrensis* population were self-pollinated for two generations to produce an inbred line (Souza et al. 2023) that was used for genome sequencing. Seed of *C. bonariensis* was collected in California, USA from an agricultural production setting and transferred to Colorado, USA. At Colorado State University, seeds were grown from each species under greenhouse conditions. A single flowering plant from each species was selected from each species for genome sequencing. From each plant, 4 g of fresh leaf tissue was sampled for PacBio HiFi library preparation and 2 g of fresh leaf tissue was sampled for Hi-C library preparation. In each case, tissue was flash frozen in liquid nitrogen and stored at −80 °C until use. The same individual plants were used to harvest 4 g of fresh tissue that was immediately shipped on damp paper towels at 4 °C for Bionano library preparation. Finally, a group of five other plants from each species was grown and used to sample various tissues (root, leaf, stem, and flower) that were flash-frozen for RNA extraction. All tissues were shipped to the Corteva Agriscience Genome Center of Excellence in Johnston, Iowa, USA, for library preparation and sequencing.

### Library Preparation and Sequencing

Library preparation and sequencing methods used here are fully described by Lemas et al. (2025). Briefly, Hi-C libraries were generated from flash-frozen tissue by cross-linking DNA with formaldehyde and digesting with DpnII. The Hi-C libraries were sequenced on an Illumina Novaseq 6000. Total RNA was extracted from the pooled tissue sample and used to generate cDNA libraries of mRNAs through poly-A selection. The cDNA libraries were used to generate a full-length transcript sequencing (Isoseq) library for each species that was sequenced on a Pacific Bioscience (PacBio) Sequel IIe machine. Flash-frozen leaf tissue was used to generate PacBio HiFi libraries and sequenced on a Sequel IIe. Finally, fresh tissue, stored at 4 °C was used to extract ultra-high molecular weight (uHMW) DNA. This uHMW DNA was labeled with DLE-1 and stained before being separated, imaged, and digitized to generate direct label and stain (DLS) molecules for Bionano.

### Genome Assembly

Methods used for genome assembly and annotation are fully described by Lemas et al. (2025) and Raiyemo et al. (2025). Briefly, HiFiasm (v0.16.1) was used to generate the primary contig assemblies for each species (Cheng et al. 2021). Contigs shorter than 70 Kbp were discarded. For *C. sumatrensis*, only HiFi data was used in this step, and haplotypes were collapsed to produce one reference assembly.

For *C. bonariensis*, HiFi and Hi-C data were used to generate phased contig assemblies for each haplotype. For each species, DLS molecules were assembled into optical maps using the Bionano Access (v1.6.1) software (Bionano Genomics, CA, USA). These optical maps were used to assemble the contigs from each species/assembly into hybrid scaffolds with Bionano Access. In the case of *C. sumatrensis*, this resulted in a single chromosome-level assembly. For *C. bonariensis*, Hi-C reads were used to create contact maps between hybrid scaffolds by running the default settings of Juicer (v1.6) (Durand et al. 2016). Contact maps were then visualized using Juicebox (v1.11.08) and used along with synteny information between haplotypes to assemble hybrid scaffolds into chromosomes. Spacers of 100 Ns were used to represent gaps of unknown length between hybrid scaffolds within a chromosome. Any hybrid scaffolds that could not be placed into chromosomes were concatenated into chromosome 00 and separated with 100 Ns. Quast (v5.2.0) was used to calculate assembly statistics for each assembly (Gurevich et al. 2013).

### Subgenome Assignment, Genome Annotation, and Synteny Analysis

SubPhaser was used to identify kmers that are enriched in each subgenome of each species and group homoeologous chromosomes into subgenomes (Jia et al. 2022). RepeatModeler (v2.0.2) was used to generate a library of repetitive elements within each genome assembly, and RepeatMasker (v4.1.2) and BEDtools (v2.30.0) were used to generate a softmasked version of each assembly (Quinlan and Hall 2010; Flynn et al. 2020). Isoseq reads were aligned to each assembly using pbmm2 (v1.10.0) (https://github.com/PacificBiosciences/pbmm2), and isoforms were collapsed using IsoSeq3 (v3.8.0; https://github.com/ylipacbio/IsoSeq3). These collapsed alignments were used along with proteins from *C. canadensis* (Laforest et al. 2020) and *Helianthus annuus* (Badouin et al. 2017) genomes to produce a gene set for each assembly using Maker-P (v3.01.04) (Campbell et al. 2014). Putative function and subcellular location were assigned to these gene sets through alignment to several protein databases (Höglund et al. 2006; Blum et al. 2009; Jones et al. 2014; Blum et al. 2025; The UniProt Consortium 2025). Assembly and annotation completeness were assessed using BUSCO (v5.7.1) and the embryophyte_odb10 dataset (Waterhouse et al. 2018). Telomeric repeats were identified using the TeloExplorer functionality within quarTeT (Lin et al. 2023).

Phased and annotated assemblies were assessed to visualize synteny using GENESPACE (v1.3.1) between subgenomes and with the genome of *C. canadensis* (Laforest et al. 2020; Lovell et al. 2022). The species tree produced by Orthofinder (v2.5.5) was used to visualize divergence between subgenomes and species (Emms and Kelly 2019).

### Identification, Naming and Classification of Cytochrome P450 Monooxygenase Genes

To identify *cytochrome P450 monooxygenase* (*CYP*) genes within the annotated protein sequences of *C. bonariensis* and *C. sumatrensis*, the InterPro codes “IPR001128” and “IPR036396” were searched for in the annotation file. All identified proteins were submitted to the Standardized Cytochrome Nomenclature Committee (http://drnelson.uthsc.edu/CytochromeP450.html) to ensure uniformity in naming conventions. For clan classification, 518 and 585 full-length CYP450 protein sequences from *C. bonariensis* and *C. sumatrensis*, respectively, were aligned to known *CYP* protein sequences using Clustal W. A neighbor-joining phylogenetic tree was generated using MEGAX software with 1000 bootstrap replicates. The resulting tree was visualized using the ITOL9 web server (https://itol.embl.de/).

## Supplementary Material

evaf065_Supplementary_Data

## Data Availability

Assemblies and annotations for *C. sumatrensis* and *C. bonariensis* are available on Weedpedia (https://weedpedia.weedgenomics.org/) and NCBI under accession ID: JBKGAD000000000 for *C. sumatrensis* and for *C. bonariensis* (haplotype 1 accession JBMMKQ000000000 and haplotype 2 accession JBMMKR000000000). Sequencing data used in the assembly and annotation are available through NCBI under BioProjects PRJNA1162129 and PRJNA1162130 and SRA accessions SRR30692215-SRR30692216 and SRR30692292-SRR30692294 for *C. sumatrensis* and *C. bonariensis*, respectively (list of raw data and accession identifiers provided in supplementary table S7, Supplementary Material online).

## Supplementary Material

Supplementary material includes one figure and six tables. Supplementary material is available at *Genome Biology and Evolution* online.
