# Supplementary material for: Chromosome-Level Assemblies of the Allohexaploid Genomes of Conyza sumatrensis and Conyza bonariensis
Source: Genome Biol Evol. 2025 Apr 4;17(4):evaf065. doi: 10.1093/gbe/evaf065 (PMC12008746; doi:10.1093/gbe/evaf065)
Supplement: evaf065_Supplementary_Data [file evaf065_supplementary_data.zip › supplemental_figure_Conyza_genomes.docx]

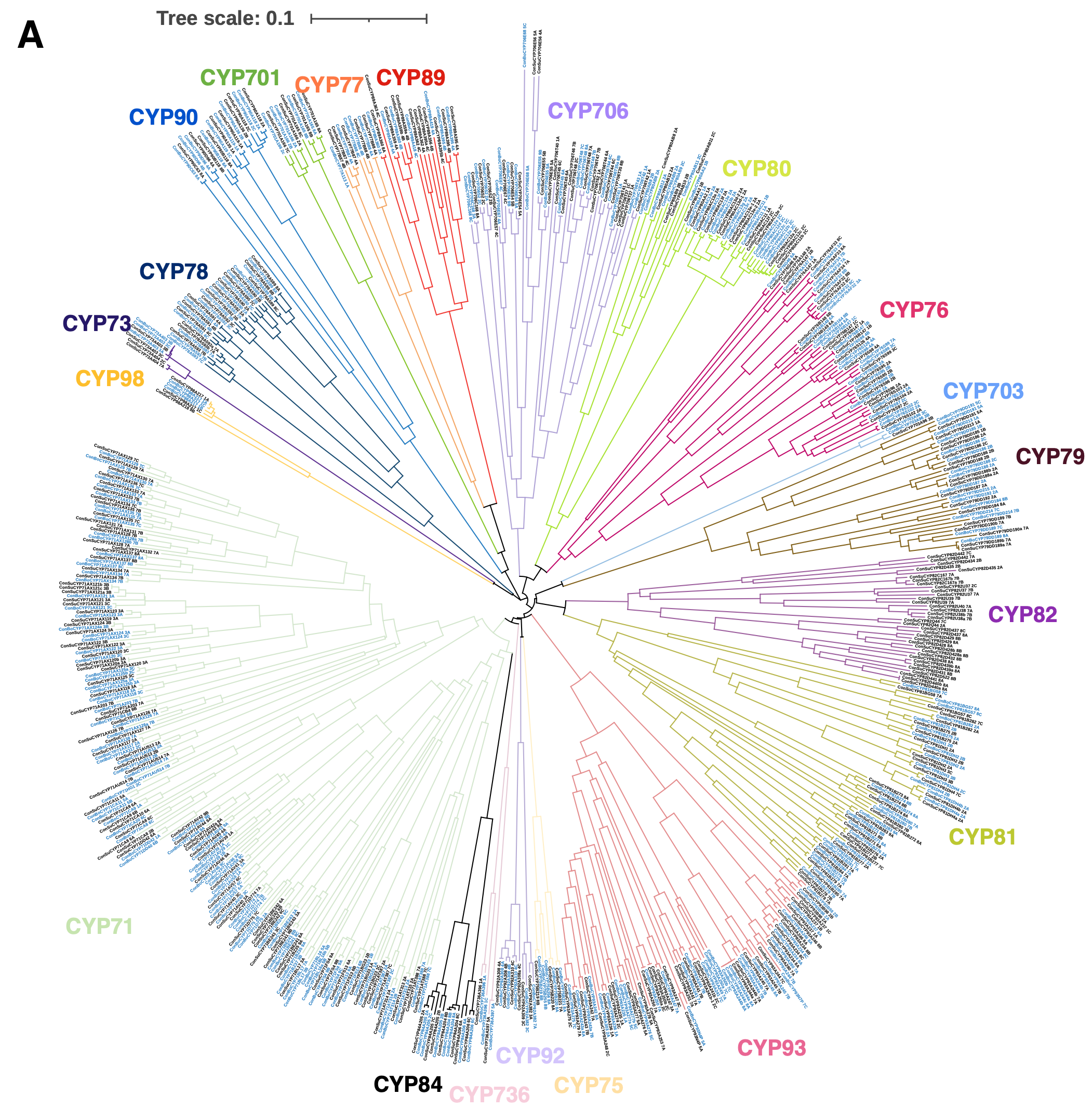


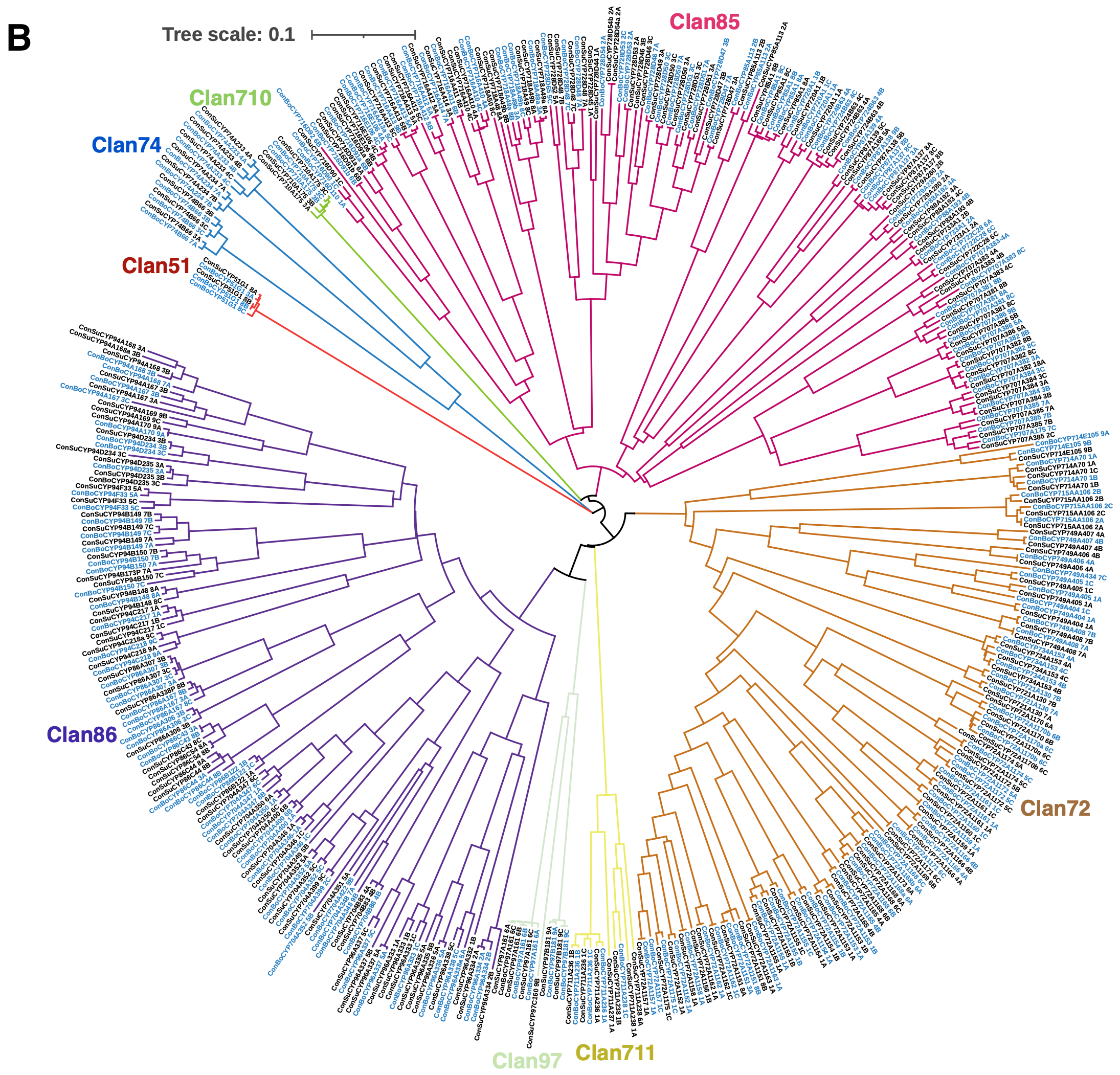


Figure 1. Classification of 518 ConSuCYP450 (*Conyza bonariensis*) and 585 ConBoCYP450 (*Conyza sumatrensis*) genes. **(A)** A-type and **(B)** non-A-type. The phylogenetic tree was constructed using the neighbor-joining (NJ) algorithm in MEGA X software, with bootstrapping performed using 1,000 replications. Different clans of P450 genes are represented by various colors in the tree. Genes from *C. bonariensis* are indicated in blue, while those from *C. sumatrensis* are shown in black.
